# Circadian Regulation in Tissue Regeneration

**DOI:** 10.3390/ijms20092263

**Published:** 2019-05-08

**Authors:** Ellen Paatela, Dane Munson, Nobuaki Kikyo

**Affiliations:** 1 Stem Cell Institute, University of Minnesota, Minneapolis, MN 55455, USA; paate001@umn.edu (E.P.); munso220@umn.edu (D.M.); 2 Department of Genetics, Cell Biology, and Development, University of Minnesota, Minneapolis, MN 55455, USA

**Keywords:** circadian rhythms, tissue regeneration, skin, intestine, hematopoiesis, ribosome biogenesis

## Abstract

Circadian rhythms regulate over 40% of protein-coding genes in at least one organ in the body through mechanisms tied to the central circadian clock and to cell-intrinsic auto-regulatory feedback loops. Distinct diurnal differences in regulation of regeneration have been found in several organs, including skin, intestinal, and hematopoietic systems. Each regenerating system contains a complex network of cell types with different circadian mechanisms contributing to regeneration. In this review, we elucidate circadian regeneration mechanisms in the three representative systems. We also suggest circadian regulation of global translational activity as an understudied global regulator of regenerative capacity. A more detailed understanding of the molecular mechanisms underlying circadian regulation of tissue regeneration would accelerate the development of new regenerative therapies.

## 1. Introduction

Earth’s 24 h rotation around its axis has influenced organismal development to be centered around cyclic patterns of day- and night-time function. Circadian systems are determined by environmental zeitgebers, or time givers, that entrain clock rhythmicity. In mammals, the most prominent zeitgebers are the onset of light and darkness, though other factors such as food intake and temperature can influence clock mechanisms [1,2]. The circadian system consists of both central and peripheral clocks. The central clock in mammals stems from a network of neurons in the suprachiasmatic nucleus (SCN); these neurons, in addition to maintaining their own cell-intrinsic clock, receive photic cues from the retina to synchronize peripheral day and night cycles throughout the body using a variety of mechanisms, including nervous system signaling, body temperature regulation, hormonal signaling, and regulation of metabolism [2,3]. Peripheral tissue circadian rhythms are synchronized by the central clock, but they also contain their own cell-intrinsic circadian rhythms [4]. As evidence, cells in culture also retain cell-autonomous 24 h rhythmicity, although synchronization is required for detection at the whole culture level [5].

In mammalian cells, peripheral clocks are maintained by two major auto-regulatory feedback loops that involve transcription of master regulator genes *Clock* and *Bmal1*. Clock and Bmal1 proteins heterodimerize and bind to enhancer E-box regions that promote transcription of thousands of genes throughout the body; 43% of protein encoding genes show circadian oscillations in expression in at least one organ [6,7,8,9]. Transcriptional targets include negative circadian master regulator genes *Per1*, *Per2*, *Per3*, *Cry1*, and *Cry2* [6,10]. The first auto-regulatory feedback loop is formed when Per and Cry proteins accumulate in the cytoplasm. Eventually, Cry/Per heterodimers translocate to the nucleus and bind the Clock/Bmal1 complex, inhibiting further *Cry/Per* transcription as well as the transcription of other genes regulated by the Clock/Bmal1 complex [7,8,11,12,13]. Cry and Per proteins are eventually ubiquitinated and degraded, allowing for another rise in Clock/Bmal1 activity [14,15].

Levels of the Clock/Bmal1 complex are regulated by a second auto-regulatory feedback loop that affects transcription of *Bmal1*. Clock/Bmal1 complexes induce expression of nuclear receptor transcriptional activators *RORα* (retinoic acid receptor-related orphan receptor α) and *RORβ*, and repressors *Rev-ErbAα* (reverse c-erbAα) and *Rev-ErbAβ*. *Bmal1* transcription is affected by competitive binding of these two nuclear receptors to Rev-ErbA/ROR response elements (RREs) in the *Bmal1* promoter region. Rev-Erbs inhibit *Bmal1* expression, while RORs promote *Bmal1* expression as essential components to stabilize circadian rhythmicity [7,8,16,17]. A variety of chromatin-modifying enzymes, kinases, phosphatases, and RNA-binding factors also modify these core master regulators to ensure circadian rhythmicity [7,8].

**Figure 1:**
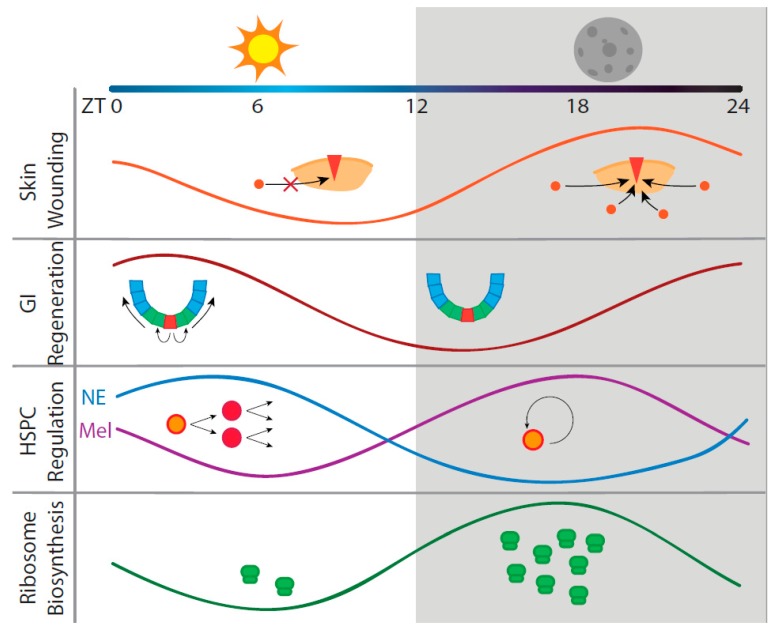
Examples of circadian interactions in regenerating systems. Circadian rhythms have been shown to impart diurnal differences in regeneration in several mouse tissue types. In skin, fibroblast migration to the site of wounding is under circadian regulation and controls wound healing efficiency [27]. In intestines, mitotic activity of intestinal crypt cells during GI damage-induced regeneration is under circadian control [28]. HSPC differentiation versus self-renewal signals are regulated by central clock norepinephrine (NE) and melatonin (Mel) secretion [29]. An understudied mechanism that may contribute to differences in a global regenerative state is fluctuations in ribosome biogenesis, which displays diurnal rhythmicity [30]. Diagrams are not drawn to scale and are meant to show general trends.

Circadian rhythms from both central and peripheral clock mechanisms have been found to influence efficacy of regeneration of many different tissues. Among the many cell types involved in regeneration, stem cells have varied circadian rhythmicity depending on differentiation state, with an extreme example being the lack of master regulator rhythmicity in pluripotent stem cells. Reflecting the current interest in stem cell biology, circadian regulation of stem cell activity has been comprehensively reviewed in recent articles [18,19]. Another widely studied area, circadian gating of cell cycle progression at multiple checkpoints, including the G1-S and the G2-M transitions, has also been extensively studied and reviewed, both in physiological tissues and in the context of carcinogenesis [20,21,22,23,24,25,26]. Therefore, in this review, we highlight circadian regulation of stem cell biology, cell cycle, and other cellular functions from the perspective of regeneration in three specific organs: skin, intestine, and blood (Figure 1). These representative tissues demonstrate time of day-dependent differences in regenerative capacity, an understudied but important contributor during wound healing. We also propose that circadian fluctuations of global translational activity may affect the regenerative capacity at any given time of day and should be taken into consideration in future studies of regeneration.

## 2. Circadian Regeneration in Three Representative Organ Systems

### 2.1. Skin Regeneration

The skin is a complex organ comprised of many different cell types. Regeneration is a coordinated effort between keratinocytes, fibroblasts, hair follicle bulge stem cells, immune cells, vascular cells, and other cells near the area of damage. Immediately after injury, signal cascades from damaged blood vessels lead to platelet activation and subsequent clotting; platelets release many growth factors to surrounding cells that assist with the tissue repair process. Inflammatory cells also infiltrate the damaged tissue and fight microbial infection while also releasing compounds, such as nitrous oxide and reactive oxygen species (ROS) [31,32,33]. After scab formation over the damaged area, nearby skin cells can begin the process of closing the wound. In the epidermis, keratinocytes and fibroblasts migrate and proliferate towards the site of injury in a coordinated manner after a series of functional changes [34,35]. These include changes in cell adhesion to allow for detachment from the basal membrane, formation of actin-rich lamellipodia for crawling towards the wound site, and upregulation of matrix metalloproteases and other proteolytic enzymes for ease of travel through the scab and wound area [33,36,37,38]. Soon after wounding, epidermal hair follicle bulge stem cells also differentiate into keratinocytes and migrate to the surface to stimulate healing [39]. In the dermis, the wound is healed through the proliferation and invasion of migrating fibroblasts and circulating multipotent fibroblast progenitor cells [33]. Each cellular response to injury in skin is highly coordinated, and efficacy of wound healing is subject to circadian influences. The circadian clock is not consistent across all skin cell types; it is more effective to consider skin circadian regulation as a collection of different but potentially coordinated peripheral clocks [40]. Three main skin cell types—fibroblasts, keratinocytes, and hair follicle bulge stem cells—and other cell types have been studied in the context of circadian rhythms in wound healing, as summarized in Table 1.

The most convincing evidence for diurnal differences in skin wound regeneration was shown with rhythmic modulation of fibroblast mobilization. In a study of mouse skin explants extracted at various time points, wounds healed significantly faster when the skin was harvested and wounded during the nighttime, or mouse active phase [27]. Through further study of synchronized fibroblast monolayer culture, the increase in healing capacity was attributed to increased efficiency of actin assembly in invading fibroblasts toward the site of wounding. Analysis of the fibroblast proteome revealed actin lamellipodia assembly was coordinated through a cell-intrinsic circadian clock [27]. High *Per2* expression correlated with increased motility and a significantly faster time to heal than when *Per2* was low [27]. This rhythmicity of wound healing efficacy also correlated with human burn healing data that showed an approximate 60% increase in the time to heal when the injury occurred during the nighttime (human resting phase) versus the daytime [27]. This study suggests that circadian rhythms may be an important variable when considering timing of wound healing treatments.

Keratinocytes, the most abundant cell type within the epidermis, interact with fibroblasts and are also influenced by circadian rhythms [46]. Human keratinocyte stem cells have cell-autonomous circadian-controlled function with regard to proliferation, differentiation, and ultraviolet (UV) response, all of which are involved in both tissue regeneration and homeostasis [41]. More specifically, differentiation-related genes are upregulated in “late night and early morning” while UV protection, DNA replication, and cell cycle genes are upregulated in “afternoon and evening” [41]. Another study found that mRNA levels of transcription factor Krüppel-like factor 9 (*KLF9*) are modulated by circadian rhythms. Daytime upregulation of cortisol due to extrinsic central clock signaling induces expression of *KLF9*, which controls cell differentiation and proliferation, adding another layer of time-dependent keratinocyte function [42]. These different layers of keratinocyte circadian regulation likely contribute to the difference in regenerative capacity based on time of injury.

Hair follicle bulge stem cells are also involved in epidermal wound repair by acutely responding to injury [39]. Natural cycling in the hair follicle niche follows a regular cycle of anagen (hair growth) and telogen (inactivity). In anagen, hair follicle bulge stem cells and their differentiated descendant germ progenitors proliferate and differentiate into transient-amplifying matrix cells (line the sides of the bulge) and hair shaft cells [44]. Bulge stem cells differentiate into keratinocytes and migrate to the site of wounding. These cells are regulated via circadian peripheral clocks. In mice, *Bmal1* expression primes a stem cell population for activation and *Per1/2* preserve a dormant population both within the stem cell bulge [43]. This study suggests a time-dependent difference in stem cell wound response.

Since all of the cell types within the hair follicle niche are involved in complex signaling interactions with other cells in the niche [47], circadian influences in one cell type likely influence other cells in the niche. This should also be taken into consideration when analyzing the regenerative capacity of bulge stem cells. Circadian clock genes are highly expressed in mouse hair germ progenitors in early anagen; *Clock* or *Bmal1* knockout delays anagen progression via prevention of germ progenitor cell cycle progression past G1 phase [44]. Epithelial matrix cells also have rhythmic oscillations in mitotic activity due to circadian gating of the G2-M phase checkpoint, with faster hair cell division and growth in the morning compared to the evening [45]. This diurnal difference led to differences in hair loss between radiation exposure in morning and evening; hair loss was more prominent in mitotically active morning injury than in the evening. This study revealed another circadian-regulated gate block to anagen progression, this time in the G2-M phase transition of epithelial matrix cells [45]. Although these studies in circadian regulation do not directly address the roles of migratory bulge stem cells in tissue regeneration, the difference in mitotic activity in other bulge niche cells based on circadian time would have an impact on their regenerative capacity in response to wounding.

### 2.2. Intestinal Regeneration

Another system that is subjected to circadian regulation of regeneration is the intestine, where the entire epithelial layer is replaced every five days [48]. Normal intestinal epithelial structure contains folded crypts of Lieberkühn, which surround villi that protrude into the intestinal lumen [49]. At the basal tip of the crypt reside crypt-base columnar cells (CBCs), the intestinal stem cells (ISCs) [50]. Progeny from CBCs becomes rapidly-proliferating transit-amplifying (TA) progenitors, which divide and move apically toward the villi. These TA cells differentiate into four different specialty intestinal epithelial lineages: Nutrient and water absorptive enterocytes (ECs), mucous-secreting goblet cells (GCs), hormone-secreting regulatory enteroendocrine cells (EECs), and Paneth cells (PCs), which travel back down to the base of the crypt and perform innate immunological and antimicrobial functions [50]. Continuous replacement of crypt cells is balanced by regulated apoptosis in the apical villi [51]. Many of the cell types within this niche are influenced by circadian rhythms, which are summarized in Table 2.

Regulation of intestinal epithelial turnover by circadian cycles has been studied for some time, most often in the context of studying the influence of light entrainment and feeding-fasting cycles to general synchronization of proliferation. Light is the determining factor in proliferation synchronization, but food administration can influence rhythmicity in the absence of light cues [58]. Other studies also described rhythmicity in crypt cell proliferation, demonstrating higher crypt cells totals during the day and higher rates of mitosis in the evening [59,60]. In a later, more mechanistic study of *Drosophila* intestine, ISCs and their differentiated lineages, with the exception of EECs, displayed circadian rhythms in normal epithelial turnover. In addition to these individual cell-intrinsic rhythms, crosstalk between cell types in this niche plays a vital role in maintenance of local synchronization. When EC rhythmicity was ablated through cell type-specific RNAi, oscillation of adjacent ISC circadian regulators was significantly reduced [52]. Since ECs are the most abundant cells on the surface of villi, they are widely exposed to environmental cues, such as ingested food, inflammatory cells, and gut microbiota [52]. This exposure of ECs could influence their crosstalk with ISCs, making epithelial turnover more complex and dependent on environmental cues.

Recent studies have discerned several mechanisms of circadian regulation in regenerating intestinal epithelia, specifically after damage from gastrointestinal (GI) diseases. Many epidemiological studies have found that these diseases are exacerbated by circadian rhythm disruption (CRD), such as jet lag, sleep deprivation, shift work, and changes in diet and physical activity [61,62,63]. Ingestion of dextran-sodium sulfate (DSS) is a commonly used approach in mice and *Drosophila* to recapitulate the epithelial damage due to GI diseases [64]. This model was used to test the impact of CRD on intestinal regeneration, comparing wild type and *Per1/2* double knockout arrhythmic mice. The knockout mice had increased susceptibility to severe damage by DSS as shown by increased necroptosis, or inflammatory cell death, of intestinal epithelial cells, and a loss of mucosal barrier GCs and signaling PCs. This was accompanied by decreased proliferation due to an upregulation of *Wee1,* a G2-M phase inhibitor, in the crypt cells [53]. This indicates that circadian rhythms are important for cell cycle control and maintenance of secretory cells in intestinal regeneration. Another study used an RNAi screen in a *Drosophila* DSS-induced colitis model and found that clock genes *per* (similar to *Per1/2* in mammals) and *cyc* (akin to *Bmal1* in mammals) are necessary for ISC mitosis and G1-S phase transition, respectively [54]. In addition, EC-specific knockdown of *per* also dampened mitotic rhythmicity in ISCs, consistent with the EC-ISC interactions in the undamaged intestine mentioned above [54]. The mitotic rhythmicity in ISCs is also under the control of PCs. A comparison between intestinal organoids (enteroids) prepared from wild type and *Per1/2* double knockout mice demonstrated that PCs preserve cyclic ISC mitosis through circadian secretion of Wnt, a stem cell self-renewal signaling molecule [55]. This rhythmic mitosis appears to be more prominent in regenerating intestinal epithelia. This is because rhythmic mitosis was not prevalent during physiological turnover in mouse intestines, whereas it was activated in injured intestines [28]. Radiation-induced GI damage displayed a clear example of time-of-the-day-dependent cell cycle control during regeneration. Mitotic activity of crypt cells peaked from ZT0-4 (light on from ZT0-12 and light off from ZT12-24) with a nadir of ZT12-16 [28]. In summary, current study of intestinal regeneration has been focused on circadian regulation of cell cycle progression.

Intestinal regeneration is likely further influenced by non-epithelial cells regulated by circadian rhythms. For example, T_H_17 cells are anti-microbial immune cells that release IL-17, inducing an increased pro-inflammatory state and infiltration of epithelial cells in colitis. T_H_17 lineage specification is controlled in a circadian manner by competitive binding between the transcription factor Nfil3 and the circadian regulator Rev-Erbα to the nuclear receptor RORγt. In the absence of light cues, the balance is shifted toward increased T_H_17 cell production, resulting in exacerbated colitis [56]. The gut microbiome population also diurnally cycles between different compositions of microbes, depending on food intake [57]. This compositional change would impact the environmental cues received by ECs throughout the day [57,65]. The complexity of all of these circadian interactions within the intestinal wall requires more study and needs to be taken into consideration when implementing treatments for GI diseases.

### 2.3. Hematopoietic Regeneration

Though it does not undergo injury in the same sense as the previous two tissues, the hematopoietic system requires constant replenishment. All blood cells throughout the body originate from a population of bone marrow (BM)-residing hematopoietic stem cells (HSCs) and hematopoietic stem and progenitor cells (HSPCs). In humans, the hematopoietic system must replenish 500 billion blood cells each day [66]. The bone marrow niche contains a variety of osteolineage cells, smooth muscle cells, endothelial cells, macrophages, adipocytes, and stromal cells that maintain and regulate HSCs and their differentiated cell types [66,67]. HSPCs undergo constant self-renewal in the bone marrow and can be divided into three subgroups: Multipotent progenitors (MPPs), which have limited self-renewal but high differentiation capacity, short-term HSCs, which have an intermediate self-renewal and differentiation capacity, and long-term HSCs (LT-HSCs), which have the highest self-renewal and bone marrow repopulation capacity [67,68]. MPPs differentiate into all blood cell types in the body through complex interactions of differentiation factors that are beyond the scope of this review [69]. All differentiated blood cell types must be continuously replenished by BM-HSPCs to maintain full body organ system and immune function. This complex network of regeneration is subject to circadian regulation and is summarized in Table 3 [70,71].

The extent to which cell-intrinsic circadian gene expression regulates hematopoiesis is differentiation-state specific. Mouse HSCs have been shown to intrinsically express some circadian rhythm genes depending on the differentiation state. After sorting for a side population (SP) of BM cells enriched for LT-HSCs, mRNA of clock genes was quantified; *Per1* levels were found to be three times higher while *Cry1* expression was lower in LT-HSCs compared to whole BM cells, suggesting that differences in intrinsic clock regulation exists in different cell populations [72]. This study did not determine rhythmicity of these clock genes, however. When the same group determined differences in rhythmic gene expression of core regulators between whole BM cells and SP cells, SP cells only showed regular oscillations in *Per2*, while BM cells displayed oscillations of *Per1*, *Per2* and *Rev-erbα* [73]. These observations suggest that HSCs and HSPCs are not subjected to regular cell-intrinsic circadian rhythm oscillations. In a later stage of hematopoiesis involving T and B cell differentiation, *Bmal1* knockout did not affect efficacy of differentiation and T and B cell function [76], despite evidence that isolated CD4+ T cells and B cells, macrophages, and dendritic cells express some core clock components rhythmically [77,78]. This acquisition of rhythmicity during hematopoietic cell differentiation is similar to the differentiation of pluripotent stem cells [18,19].

Although cell-intrinsic clock mechanisms are not prominent in HSPCs, this does not mean that these cells lack diurnal cycling in regeneration. Several studies have outlined external mechanisms that stimulate time-dependent differences in blood cell replenishment. HSCs in the BM undergo self-renewal through receptor–ligand interactions between HSC CXCR4 receptors and CXCL12 chemokine secreted from BM stromal CXCL12-abundant reticular (CAR) cells [67,79,80]. Termination of this interaction leads to HPSC egress from the marrow and subsequent differentiation. CXCL12 and HSC egress have been linked to circadian rhythms through extrinsic mechanisms. In a study of mouse HSC egress, the number of circulating hematopoietic progenitors peaked at ZT5, while the population nadir was at ZT17 [70]. This difference in HSC release from the bone marrow was attributed to secretion of norepinephrine (NE) from the sympathetic nervous system in the bone marrow. NE in the BM then induces downregulation of CXCL12 in CAR cells through elimination of Sp1, the CXCL12 transcription factor [70]. Lower CXCL12 levels after light induction thus lead to higher HSC egress and blood cell differentiation in mice. Oscillations in CXCR4 receptor expression also correlate with CXCL12 fluctuations and have been implicated in influencing the level of HSC egress [74]. Lower CXCR4 in mouse HSCs promoted daytime egress from the bone marrow in mice [74]. When human blood was tested for migrating HSPCs at different times during the day, the highest levels were found in the evening, before human resting phase, indicating humans and mice have opposite cycles of HSC egress and blood regeneration due to different active and rest cycles [74]. The mechanism for this difference between mice and humans, however, was not described.

Another study characterized diurnal differences in mouse HSPC activity through a different mechanism. NE-induced tumor necrosis factor (TNF) bursts at light and dark onset induce two peaks of different HSPC activity [29]. At light onset, NE and TNF secretion induce HSPC differentiation and egress through an increase in ROS levels in HSPCs (a characteristic of HSPC activation [81]) and by increasing vascular permeability. At darkness onset, TNF increases secretion of the nighttime neurotransmitter melatonin, which reduces HPSC ROS levels, induces self-renewal of LT-HSCs, and restricts vascular permeability [29]. This balance between HSPC self-renewal and differentiation is essential to maintain a healthy population of blood cells. Another stress hormone has also been implicated in circadian HSPC regulation. Corticosterone undergoes circadian patterning and peaks at the onset of darkness in mice, reaching a nadir at dawn [75,82]. This diurnal patterning helps to maintain HSPC homeostasis through interactions with CXCL12; high corticosterone levels decrease HSPC repopulation capability in the BM and lead to a higher proportion of circulating HSPCs, while chronically low corticosterone levels had the opposite effect [75]. Collectively, these studies underscore the significance of timing as a factor influencing the outcome of regenerative therapies. For example, in mice, extraction of enriched bone marrow populations should be done in the evening during HSPC self-renewal, while isolation of migratory HSPCs in the blood stream would be more efficient during the daytime.

## 3. Circadian Translation May Be a Factor in Regeneration

Many of the mechanisms presented here used circadian transcriptome analysis as one of the main strategies for measuring circadian regulation from either cell-intrinsic clock regulators or induction from extrinsic signals. Rhythmic transcription does not always lead to rhythmic translation, however. In a study of protein accumulation in the liver, about half of the proteins involved in rhythmic morning and evening accumulation could not be attributed to rhythmic mRNAs, indicating circadian rhythmicity can be affected by post-transcriptional systems [83]. Circadian rhythmic influences have been demonstrated in a variety of post-transcriptional processes within the cell, including RNA splicing, stability, and miRNA regulation [84,85,86]. These relationships between circadian rhythms and post-transcriptional mechanisms have not yet been fully characterized as a global mechanism for circadian regulation, but they are likely to be impactful in regenerating systems [86]. The circadian influence these post-transcriptional modifications have, however, is likely impacted by specific properties of each gene being expressed. For this reason, we would like to emphasize a different mechanism of circadian regulation, global fluctuation of translational activity, which could influence overall regenerative capacity in any tissue.

Because the processes of regeneration, including cell division, differentiation, and cellular mobility, require the production of proteins not always present in the cell, rhythmic translational activity is likely to be globally important in the context of organ regeneration. The amount of total protein synthesis in rat SCN neurons displayed diurnal differences, with peak translational activity at night between ZT22-0, though the study is contested [87,88]. The difference in translational rate at the peak was close to 1.5 times the rate at the nadir [87]. In *Drosophila* pacemaker neurons, ribosomal association with clock-associated transcripts showed peaks at midday and midnight, indicating a circadian influence on translational timing [89]. These studies suggested fluctuating global translational activity as an important factor to consider for efficiency of time-dependent regeneration.

This idea is supported by several other studies that couple specific components of translational machinery to circadian rhythmicity. For example, in mouse liver, the number of ribosomal mRNAs incorporated into polysomes, an indirect measurement of translation, was higher at night than in the morning, although the levels of the total ribosomal mRNA remained unchanged [30]. Consistently, ribosomal protein translation and assembly occurred at night, indicating a higher capacity for protein synthesis during mouse active hours [30]. This was recapitulated in another study of mouse liver with ribosomal profiling, where 150 non-oscillating transcripts were found within polysomal fractions in a circadian manner. This showed a peak in polysome association of protein biosynthesis machinery genes from ZT10-16 [90] A similar phenomenon was found in the plant *Arabidopsis thaliana*, where ribosomal protein mRNAs in polysomes peaked at night. Several peaks in ribosome loading at different times of the day were present; analysis of these polysomal mRNA transcripts showed a bias towards proteins that serve a specific function at that time of day, such as photosynthetic complexes at midday (ZT6) [91]. This global trend of preferential translational activity depending on the time of day may have an impact on regenerative capacity when determining disease treatments. There may be certain times where regenerative success is more likely, depending on the cellular translational activity.

Several other circadian mechanisms have been shown to directly modulate translational activity. In mice, the Bmal1 protein acts as a translation factor in the cytoplasm through rhythmic associations with translational machinery. When phosphorylated in a circadian manner by S6 protein kinase 1 (S6K1), an effector of mammalian target of rapamycin (mTOR), Bmal1 associates with the mRNA cap-binding complex to assist in translation initiation [92]. This has been shown experimentally in rats undergoing simulated “shift work”; disruption of the rest cycle distinctly changed protein synthesis markers in the prefrontal cortex, which could explain impaired waking function [93]. In *Neurospora crassa*, another mechanism of translational regulation was found through circadian modulation of the mitogen-activated protein kinase (MAPK) pathways [94]. MAPK proteins were found to rhythmically phosphorylate eukaryotic elongation factor-2, which is a key component in translation elongation; this effect was ablated with loss of core circadian components [94]. We feel these mechanisms of circadian regulation (summarized in Table 4) are understudied in regeneration and present an intriguing question for further exploration.

## 4. Conclusions

Regeneration is often studied at the cellular level, with specific cell–cell interactions inducing change through expression of specific proliferation and differentiation factors. Regeneration can also be studied through a global physiological lens, where the large-scale factors of metabolism, hormonal signaling, and environmental cues can impact the regenerative outcome. We believe that abundance of ribosomes and translational activity can be partnered with these big picture factors of regeneration as well, due to the global cellular requirement for protein synthesis. Because circadian mechanisms can act on many of these different levels, it is important to consider all of the possible influences when studying regeneration. Although circadian rhythm interactions at the cellular level seem very easily detectable, there is always a physiological undercurrent that may influence the outcomes, such as high metabolic activity or low ribosomal biosynthesis, which can create a favorable or unfavorable environment for regeneration. Dissecting these multilevel, interacting circadian influences will be a challenging but rewarding field of study that will hopefully elucidate ideal timing for regenerative therapies.

## Figures and Tables

**Table 1:** Circadian regulation of skin cells.

Cell Type	Model	Circadian Regulation Mechanism	Conclusions	Ref.
Mouse fibroblasts	Skin explants, in vivo, synchronized culture	Cell-intrinsic—actin lamellipodia formation	Correlation between high *Per2* in night-time and increased mobilization and wound healing	[27]
Human keratinocytes	Synchronized keratinocyte culture, in vivo competition in nude mice	Cell-intrinsic differentiation or proliferation response	Transcriptome: high differentiation in early morning, high proliferation in evening	[41]
	Epidermal biopsies, neonatal keratinocyte culture	Cortisol-induced *KLF9* expression	High *KLF9* in morning, associated with increased differentiation	[42]
Mouse hair follicle bulge stem cells	In vivo reporter mouse	Cell-intrinsic *Bmal1* and *Per1/2* regulation	Circadian cycling maintains homeostasis of stem cell population	[43]
Mouse hair germ progenitors	In vivo mouse dorsal skin	Cell-intrinsic regulation of cell cycle	Clock genes regulate G1-S phase transition in hair germ	[44]
Mouse hair epithelial matrix cells	In vivo mouse dorsal skin–radiation hair loss	Cell-intrinsic regulation of cell cycle	More hair loss in morning during high mitotic activity, clock genes regulate G2-M phase transition	[45]

**Table 2:** Circadian intestinal stem cell niche interactions in regeneration.

Cell Type	Model	Circadian Regulation Mechanism	Conclusions	Ref.
*Drosophila* crypt cells	In vivo physiological turnover, circadian knockouts	Intercellular niche signaling from ECs to ISCs	ISC rhythmicity influenced by ECs	[52]
Mouse crypt cells	In vivo DSS-induced colitis, circadian knockouts	Intercellular signaling	Arrythmicity leads to more severe colitis through loss of crypt cells and G2-M inhibition	[53]
*Drosophila* crypt cells	In vivo RNAi screens in DSS-induced colitis	Intercellular signaling of circadian factors	*per* transcript peaks ZT12-18, induces peak ISC mitosis at dawn, local signaling of clock components essential for G1-S phase	[54]
Mouse Paneth cells in crypt	Enteroid culture	Wnt secretion from PCs	PCs are necessary for pacemaker circadian regulation of ISC cell division	[55]
Mouse crypt cells	Radiation-induced GI syndrome in vivo and in enteroids	Circadian mitotic schedule in response to injury	Mitotic activity peak ZT0-4 and nadir ZT12-16	[28]
Mouse Intestinal T_H_17 cells	In vivo intestine and colon	Balance of T_H_17 differentiation through competing *Rev-Erb* and *Nfil3*	T_H_17 cells are pro-inflammatory, *Nfil3* and *Rev-Erb* necessary to balance T_H_17 population—disruption exacerbates GI diseases	[56]
Mouse gut bacterial cells	Gut microbiome	Cyclical fluctuations in microbial population	Food intake timing can influence microbial effect on intestines	[57]

**Table 3:** Circadian regulation of hematopoietic stem and progenitor cell (HSPC) activity.

Cell Type	Model	Circadian Regulation Mechanism	Conclusions	Ref.
Mouse BM SP (LT-HSC enriched)	Isolated BM cells	Cell-intrinsic circadian clock in SP LT-HSCs	LT-HSCs show high *Per1* and low *Cry1* expression compared to total BM cells, only *Per2* is oscillating in SP cells, irregular circadian clock in LT-HSCs	[72,73]
Mouse BM and blood cells	Isolated BM and blood cell culture	NE and CXCL12 signaling from BM nerve and CAR cells	Circulating HSPC number peaks at ZT5 and shows nadir at ZT17, NE from nerves downregulates CAR CXCL12 and induces HSC egress	[70]
Circulating human and mouse HSCs and HSPCs	Peripheral blood isolation at different time-points	CXCR4 and CXCL12 circadian regulation in the BM	Mice and humans showed opposite egress patterns, human egress peak in the evening	[74]
Mouse BM and circulating HSCs	In vivo, cultured BM and circulating HSCs	NE induced TNF bursts at light and dark onset	NE and TNF bursts at light onset induce HSPC differentiation and egress, while TNF and melatonin bursts at dark onset induce HSC self-renewal	[29]
Mouse BM and circulating HSPCs	In vivo in WT and Corticosterone deficient mice, cultured BM, and circulating HSPCs	Corticosterone modulation of BM CXCL12 secretion	Corticosterone peaks at dawn and downregulates CXCL12. Rhythmicity essential to balance HSPC egress and self-renewal	[75]

**Table 4:** Circadian regulation of translational activity.

Model	Circadian Regulation Mechanism	Conclusion	Ref.
Mouse liver	Ribosomal mRNA association with polysomes	More ribosome subunit synthesis and assembly during nighttime.	[30]
Mouse liver	Ribosomal mRNA association with polysomes	Peak polysome association with ribosome transcripts ZT10-16, 150 non-oscillating transcripts had preferential translational timing based on function	[90]
*Arabidopsis thaliana* seedlings	Ribosomal mRNA association with polysomes	Proteins with daytime or nighttime function preferentially associate with ribosomes at that time of day. Ribosomal mRNAs bound to polysome at night	[91]
Mouse embryonic fibroblasts	Bmal1 association with translation machinery	S6K1-mediated phosphorylation of Bmal1 promotes its binding to mRNA cap-binding complex and increased translation	[92]
*Neurospora crassa*	Circadian MAPK phosphorylation of elongation factors	MAPK factors rhythmically phosphorylate eEF-2, increasing translation efficiency	[94]

## Abbreviations

SCN	Suprachiasmatic nucleus
ROR	Retinoic acid receptor-related orphan receptor
Rev-ErbA	Reverse c-erb A
RRE	Rev-ErbA/ROR response element
ROS	Reactive oxygen species
UV	Ultraviolet
CBC	Crypt-base columnar cell
ISC	Intestinal stem cell
TA	Transit-amplifying
EC	Enterocyte
GC	Goblet cell
EEC	Enteroendocrine cell
PC	Paneth cell
GI	Gastrointestinal
DSS	Dextran–sodium sulfate
CRD	Circadian rhythm disruption
RNAi	RNA interference
ZT	Zeitgeber time
BM	Bone marrow
HSC	Hematopoietic stem cell
HSPC	Hematopoietic stem and progenitor cell
MPP	Multipotent progenitor
LT-HSC	Long-term hematopoietic stem cell
SP	Side population
CAR	CXCL12-abundant reticular
NE	Norepinephrine
TNF	Tumor necrosis factor
MAPK	Mitogen-activated protein kinase
